# Global evidence of non-pyramidal and uniform ratios of animal diversity across terrestrial trophic levels

**DOI:** 10.1098/rspb.2025.2335

**Published:** 2025-11-26

**Authors:** Luis F. Camacho, Miguel B. Araujo

**Affiliations:** ^1^ Museo Nacional de Ciencias Naturales, Consejo Superior de Investigaciones Científicas, Madrid 28006, Spain; ^2^ Mediterranean Institute for Agriculture, Environment and Development (MED), and Global Change and Sustainability Institute (CHANGE), Universidade de Évora, Évora 7002-554, Portugal; ^3^ Theoretical Sciences Visiting Program, Okinawa Institute of Science and Technology Graduate University, Onna 904-0495, Japan

**Keywords:** animal, biodiversity, consumers, food web, macroecology, niche

## Abstract

Thermodynamics imposes a well-established pyramidal distribution of energy availability across trophic levels, but whether species richness follows the same pattern remains unclear. In this study, we examined species richness across trophic groups for all known terrestrial tetrapod and arthropod species, representing over 90% of Earth’s terrestrial animal diversity. By categorizing species into fundamental trophic levels, we found that 46% are primary consumers (feeding on plants), 43% are higher-level consumers (feeding on primary consumers) and 11% are mixed consumers (generalists). Further analysis of global community trophic structures in mammals and birds uncovered a consistent ratio of species richness across trophic levels, independent of geographical location or total species richness. We propose that this non-pyramidal distribution of diversity arises from higher ecological differentiation among species at higher trophic levels, which may offset their greater extinction risk associated with smaller populations. This process could generate uniform trophic structures if primarily driven by mechanics intrinsic to trophic positions and their interactions rather than specific environmental characteristics. Such intrinsic mechanisms may ultimately influence diversity across trophic levels and the eco-evolutionary dynamics structuring ecological networks.

## 1. Introduction

Ecosystems operate on principles of energy transfer, making them intrinsically governed by the laws of thermodynamics [1,2]. The principle of conservation, or the first law of thermodynamics, is key to understanding the flow of energy through the ecosystem. It explains how solar energy is captured through photosynthesis, converted into biomass or used to perform work, and subsequently transferred across trophic levels via herbivory and predation. The second law, concerning entropy, explains the energetic losses at each trophic transfer, resulting in less energy flowing at higher trophic levels compared with the lower levels [3]. Thermodynamic principles therefore underpin the predictable organization of energy within food webs, influencing the evolution of trophic species niches as individuals compete for energy within their respective trophic level [4,5]. While thermodynamics is essential for understanding energy flow and distribution in ecosystems, it does not determine the eco-evolutionary processes governing the distribution of species richness across trophic levels [1,6,7]. This distinction is crucial, as it highlights the dynamic interplay between the physical constraints imposed by energy systems and the biological complexities of evolution and species interactions. It underscores the need for an integrated eco-evolutionary approach to study ecosystem organization and biodiversity [7].

Research on the trophic structure of species richness has traditionally relied on empirical food web data or dietary composition data of species from specific sites or aggregated across several locales. These analyses have often revealed a pyramidal distribution of species richness across trophic levels [8,9]. However, deviations from this pyramidal structure have also been documented, including instances of non-pyramidal ‘squared’ structures and even inverted pyramids, which call into question the generality of the pyramidal stratification of species richness (for a review, see table 1) [9]. Furthermore, identifying general patterns is complicated by the heterogeneity of the taxa analysed and inconsistencies in trophic classification schemes [24]. This variation hinders meaningful ecological comparisons across studies and obscures the underlying drivers of structural variation in trophic stratification of species richness [9]. To determine whether general patterns exist—and whether they reflect broader ecological principles—a systematic investigation of species richness patterns across trophic levels and biogeographical regions is essential. A critical aspect of such research involves analysing as many taxa as possible to encompass the full range of species involved in the trophic networks. Given the paucity of comparable food web data at a global scale, an alternative approach involves the study of inferred trophic structures within regional species pools [25]—commonly referred to as metawebs. Metawebs represent the set of potential interactions among species occurring in a given region, inferred from known feeding preferences, allometric relationships and/or co-occurrence patterns [26–28].

**Table 1.** Summary of studies examining the trophic structure of species, based on the percentage of predatory (as opposed to prey) species reported in each study. Green triangles indicate pyramidal trophic structures (<40% predatory species), red inverted triangles indicate inverted trophic pyramids (>60% predatory species) and yellow squares indicate uniform trophic structures (40–60% predatory species). Studies are ordered by the number of food webs or communities analysed (*n*).

study	taxon	realm	region	*n*	mean % of predator species in community
[8]	invertebrates	terrestrial and marine	worldwide	389	31 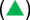
[10]	invertebrates	freshwater	Britain and North America	95	26 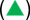
[11]	invertebrates	terrestrial and marine	worldwide	95	68 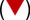
[9]	mostly invertebrates	terrestrial and marine	worldwide	72	35 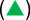
[12]	mostly invertebrates	terrestrial and marine	worldwide	60	68 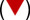
[13]	invertebrates	streams	New Zealand	14	37 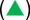
[14]	lepidoptera and parasitoids	terrestrial	worldwide	11	6 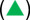
[15]	mostly invertebrates	bromeliads	Central and South America	8	26 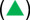
[16]	invertebrates	grasslands	Alaska and Ohio	5	48 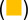
[17]	invertebrates	trees	Britain and South Africa	4	44 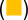
[18]	insects	grassland	Michigan	1	37 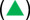
[19]	invertebrates	grasslands	Ohio	1	41 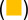
[20]	invertebrates	forest	Borneo	1	44 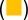
[21]	vertebrates and invertebrates	desert	California	1	8 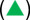
[22]	invertebrates	*Cytisus scoparius*	England	1	84 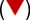
[23]	lepidoptera and parasitoids	terrestrial	England	1	69 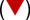

Here, we examine the global stratification of species across trophic levels in terrestrial environments by analysing the diets of all known species of terrestrial tetrapods and arthropods, encompassing over 90% of Earth’s recognized diversity of terrestrial animals (electronic supplementary material, table S1; statement supported by data from [29]). Additionally, we investigate the biogeographical distribution of trophic levels among all recognized species of terrestrial tetrapods, building on a framework that identifies six distinct community trophic structures (analogous to trophic biomes) [30] (electronic supplementary material, figure S1). These community-level trophic structures are derived from species range and diet data, allowing assessment of regional variation in the relative proportions of species belonging to different feeding guilds across ecosystems characterized by varying productivity levels [30–33] (electronic supplementary material, figure S1). While guilds provide valuable insight into energy transfer pathways, they do not directly represent the hierarchical energy organization inherent to trophic levels [34]. To bridge this gap, we compared the stratification of species richness across these six community trophic structures, assessing whether systematic variation in energy pathways is accompanied by a reorganization of trophic-level hierarchy. For this purpose, we applied a strictly energetic definition of trophic levels, originally formulated by Lindeman [34], which does not distinguish between the consumption of live and dead organic matter. In this framework, trophic levels are defined by the sequence and direction of energy flow, such that any consumption of non-autotrophic biomass is treated as higher-level interaction. Thus, we categorized species into three fundamental consumer resource levels: primary consumers (e.g. herbivores and plant detritivores); higher-level consumers (e.g. predators, scavengers and fungivores); and mixed consumers (e.g. trophic omnivores). This classification captures the core energetic structure of species assemblages and provides a foundation for examining variation in trophic-level architecture across biogeographical contexts [35].

## 2. Results

**Figure 1.**
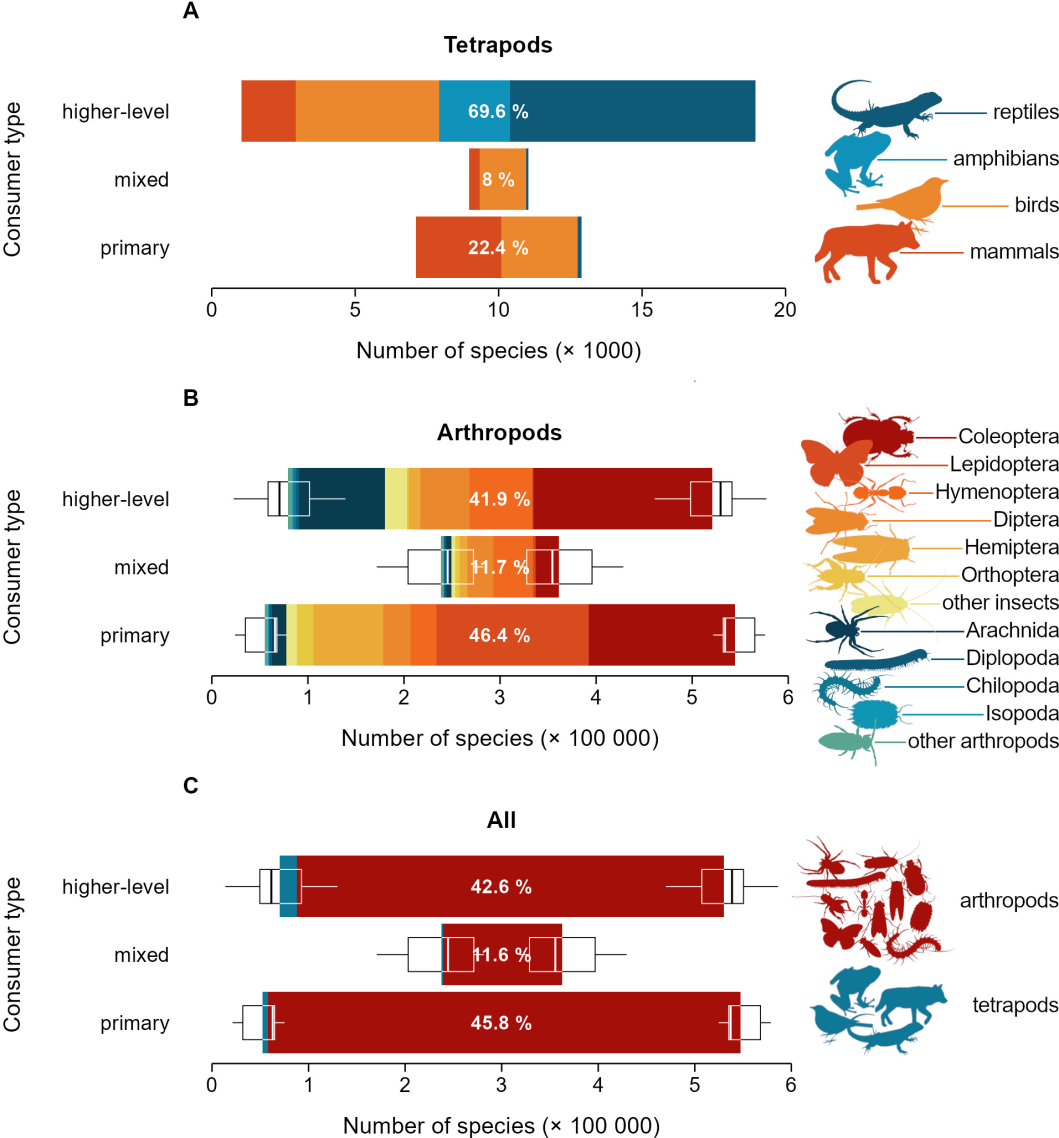
Number of species across trophic levels for the world’s tetrapods (A), arthropods (B) and both (C). Boxplots show the range of uncertainty in species numbers assigned to each trophic level. Values in white show the percentage of species in each trophic level. For specific numbers per taxon, see electronic supplementary material, table S2.

Terrestrial tetrapods display a markedly inverted trophic pyramid of species richness, with higher-level consumers comprising 69.6% of species, followed by mixed consumers at 8.0% and primary consumers at 22.4% (figure 1A). This pattern is largely driven by reptiles and amphibians, where higher-level consumers make up 97% and 100% of species, respectively (electronic supplementary material, table S2). Birds also display an inverted trophic structure, though with more heterogeneous stratification: higher-level consumers account for 54% of species, while primary consumers constitute 29% (electronic supplementary material, table S2). Mammals, on the other hand, exhibit a slightly pyramidal structure, with higher-level consumers comprising 36% of species and primary consumers 57% (electronic supplementary material, table S2). In contrast, terrestrial arthropod species display a relatively uniform trophic stratification (figure 1B), and—owing to their disproportionately high diversity—ultimately shape the global trophic ‘pyramid’ of species into a square, with higher-level consumers making up 42.6% ± 0.02% (95% CI) of species, followed by mixed consumers at 11.6% ± 0.02% (95% CI) and primary consumers at 45.8% ± 0.01% (95% CI) (figure 1C). While the shape of the trophic pyramid varies considerably across arthropod orders, a distinctly pyramidal structure characterizes only three of the major insect orders: Hemiptera, Lepidoptera and Orthoptera. Moreover, a low proportion of trophic generalist species is almost universal across tetrapod and arthropod taxa (electronic supplementary material, table S2).

**Figure 2.**
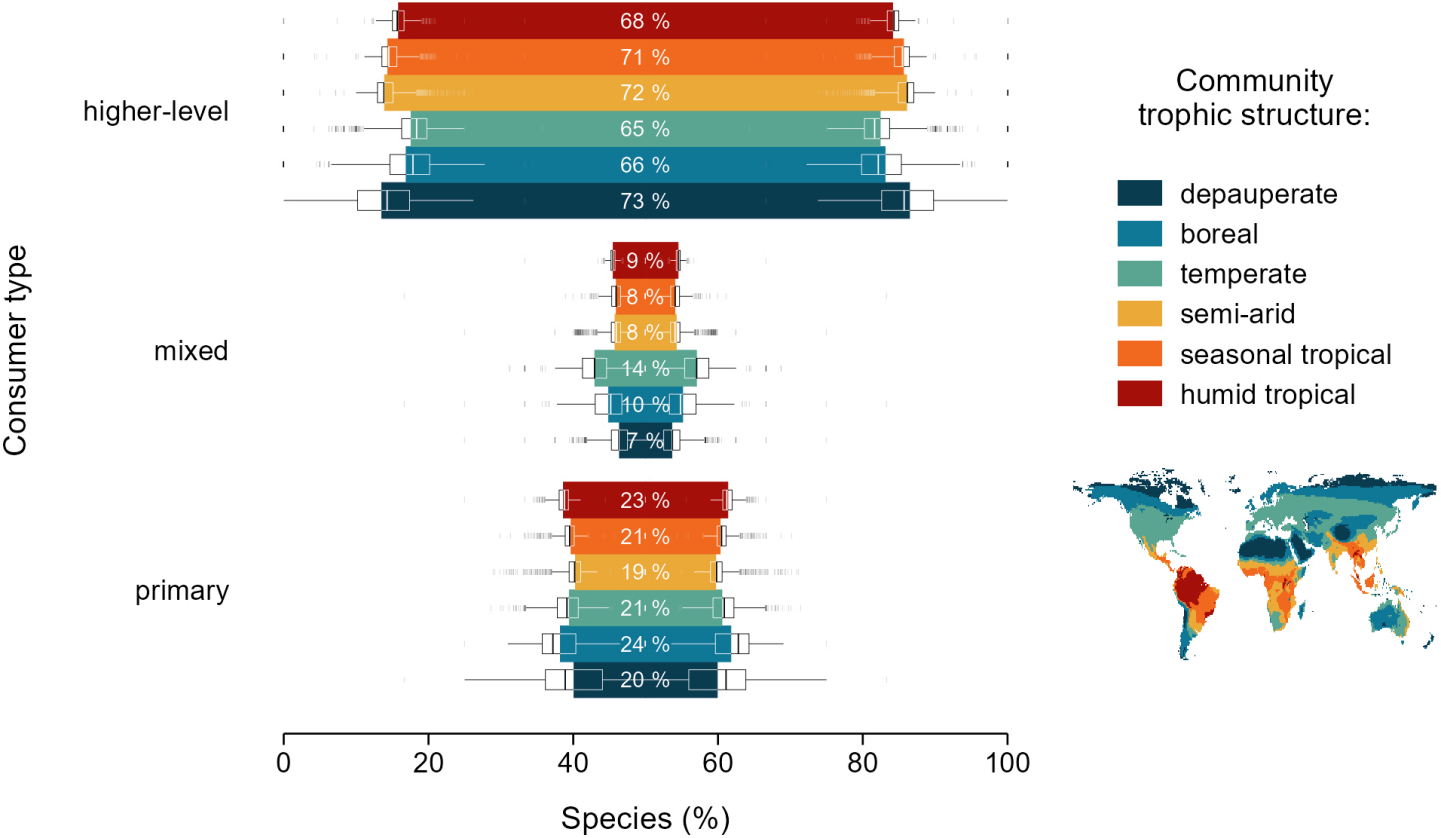
Average percentages of bird, mammal, reptile and amphibian species of each trophic level per community trophic structure across 1 degree pixels on the global map. White numbers show the average values and boxplots show the distributions of values across pixels. Graphic based on the global characterization of community trophic structures from [30].

The examination of species richness across both functional (trophic) and geographical spaces was limited to tetrapods (i.e. mammals, birds, reptiles and amphibians) owing to the availability of comprehensive range maps for these groups. Remarkably, our analysis revealed a consistent ratio of species richness in the trophic level distribution of terrestrial tetrapods across the six community trophic structures globally (figure 2). These community-level trophic structures arise from differences in the proportions of species in different guilds occurring across 1 degree pixels globally [30–33] (electronic supplementary material, figure S1). However, when attempting to classify pixels of the six community trophic structures based on the proportion of species in different trophic levels (rather than the proportion in different guilds, as originally defined), a discriminant analysis showed only a 6.6% improvement over random chance (electronic supplementary material, table S3). This finding suggests a significant degree of homogeneity across community trophic structures in the ratio of species in each trophic level, where higher-level consumers make up 68.13% ± 0.08% (95% CI) of species, followed by mixed consumers at 10.45% ± 0.04% (95% CI) and primary consumers at 21.42% ± 0.06% (95% CI). A similar degree of regularity between trophic structures emerged when analysing mammal and bird communities separately (electronic supplementary material, figure S2 and table S3).

**Figure 3.**
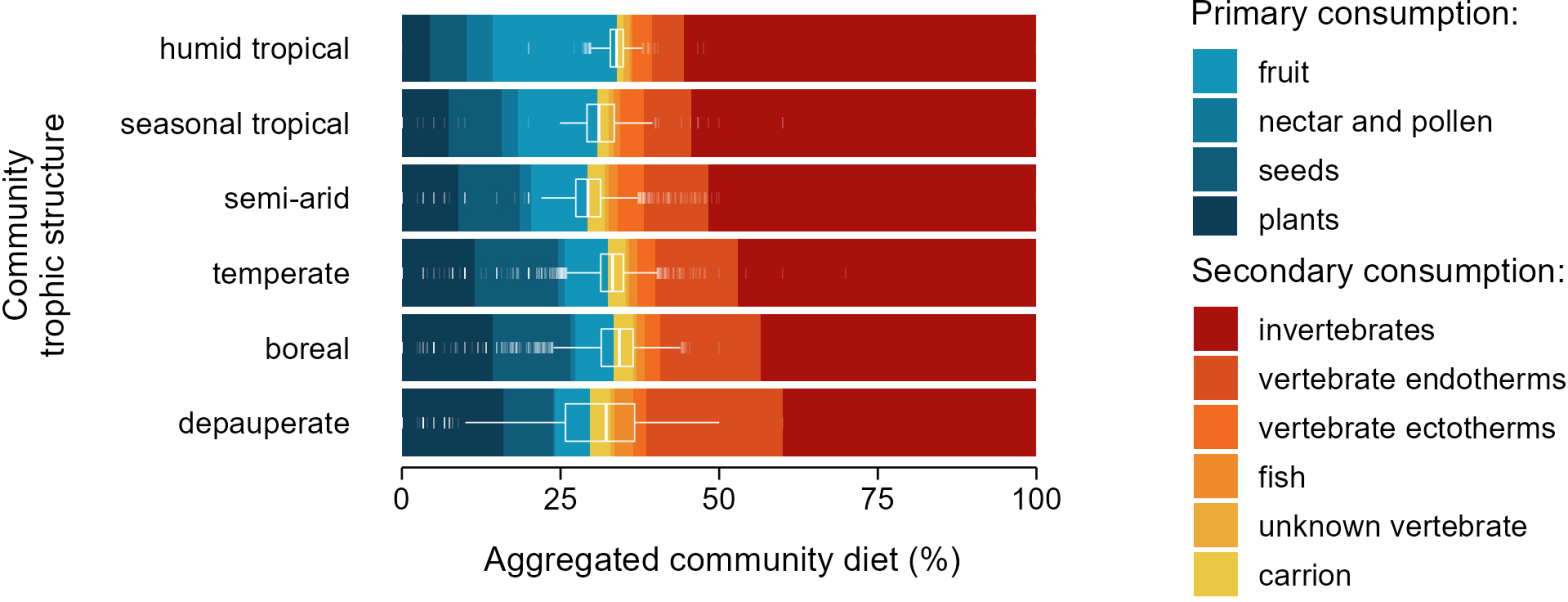
Contributions of dietary items to the aggregated diets of bird and mammal communities across 1 degree pixels on the global map, with boxplots showing the diet range between primary and higher levels per pixel. Graphic based on global characterization of community trophic structures from [30].

In a subsequent analysis, detailed dietary data for birds and mammal species enabled us to characterize the six trophic structures not by the proportion of species richness from each trophic guild but by the types and proportions of items consumed across species. Despite anticipated variations in species’ diet and their proportional influence on the aggregated community diet within each trophic structure, the relative contributions of primary and higher-level consumption to the aggregated community diet remained remarkably consistent (figure 3). Indeed, trophic structures had virtually no explanatory power in differentiating communities based on the percentage of primary versus higher-level consumption, with an *R*^2^ of just 0.06. Across all trophic structures, primary consumption consistently accounted for 31.79% ± 0.06% (95% CI) of the aggregated community diet. Similar patterns of regularity were found in separate analyses of the communities of birds (*R*^2^ = 0.08) and mammals (*R*^2^ = 0.04), where primary consumption made up 28.32% ± 0.05% (95% CI) and 42.54% ± 0.08% (95% CI), respectively, of the aggregated community diet (electronic supplementary material, figure S3).

## 3. Discussion

In contrast to our finding of a global non-pyramidal stratification of species richness across all tetrapod and arthropod groups, local trophic structures have often, though not universally, been characterized by a pyramidal structure [9,24]. One potential explanation for this discrepancy lies in differing conceptualizations of trophic levels. In our study, we adopted a strictly energetic definition of trophic levels, as originally proposed by Lindeman [34], which treats all consumption of non-autotrophic material—whether living or dead—as higher-order consumption. Under this framework, trophic levels reflect the steps and direction of energy flow that are ultimately governed by the thermodynamic principles of transformation and loss. In contrast, most empirical food web studies—particularly those focused on invertebrate communities—tend to classify detritivores, scavengers, fungivores and even omnivores as herbivores [8,10,11]. Other studies take an ecological-network approach, assigning species to basal, intermediate or top trophic positions independently of their functional role as herbivores or predators [9,12]. These differing approaches to trophic classification ultimately hinder direct comparisons of trophic structures across studies.

A direct comparison of trophic patterns between studies is further limited by differences in the taxon or ecosystem analysed. Trophic structures are an emergent property of species interactions, and, as such, a characterization of species ratios across trophic levels should ideally account for all members participating in the ecological network being analysed (see details on this matter pertaining to our study in §3a). The choice of focal taxon or the taxonomic bias inherent to any sampling method will inevitably shape the reported trophic structure by focusing on different vertical or horizontal sections of the trophic structure. For instance, we show that trophic levels are not evenly represented across taxa. Thus, study designs that skew the representation of a particular taxon (e.g. because of data availability or studied ecosystem) can potentially introduce biases in species richness across trophic levels, ultimately limiting the comparability of trophic structures across studies.

Beyond conceptual and taxonomic factors, discrepancies between studies may reflect a more interesting pattern of ecological scaling, which is particularly pertinent to our findings given their broad spatial scale. Previous analyses have suggested that the ratio of species at higher trophic levels tends to increase with spatial scale [8]. Local food web structures reflect the pool of available species at any given location—a subset of the broader regional species pool. Reduced species counts at higher trophic levels may result from sparse population densities, leading to subsampling of predator species [36]. On the other hand, at broader geographical scales, especially when entire species ranges are used to characterize assemblages of overlapping species, community trophic structures tap into the more extensive regional species pool [27,28]. This divergence between scales reflects two interconnected ecological domains, as highlighted by Rossberg [37]: on the one hand, population and meta-community dynamics shaping species occupancy and interactions at local scales; on the other hand, eco-evolutionary processes influencing species’ geographic distributions, ranges sizes and diversification across trophic groups and biogeographical regions [36,38–41]. From this perspective, the global trophic structure we describe can be interpreted as an emergent property—an evolutionary aggregate of the outcomes shaping Earth’s terrestrial biodiversity across space, time and trophic organization.

### (a) Mechanisms influencing the shape of trophic structures

While our analysis covers over 90% of terrestrial animal species, the extent to which the observed non-pyramidal trophic structure is generalizable to all terrestrial consumers depends on the trophic composition of taxa not included in our dataset. For instance, our estimate of primary consumer richness does not account for fungi, which serve as the main resources for many secondary consumer arthropods included in our analysis. Such saprophagous fungi represent a fraction of the approximately 120 000 described species of fungi [42,43], and are unlikely to substantially alter the shape of the observed trophic pattern derived from over 1 million species. A non-pyramidal trophic structure of terrestrial consumers may hold even when considering understudied taxa that are likely to include many undescribed species. For instance, global diversity of fungi has been estimated at 2.2–3.8 million species [42], and Nematoda at least 1 million species [44], both of which include a variety of trophic roles including primary and higher-level consumer species [43,45]. Most notably, insects are estimated to comprise at least 6.8 million species, including a disproportionately high number of parasitoid wasps [14,46–48], which would render the arthropod trophic structure even more top-heavy than currently observed. Ultimately, the full characterization of the global trophic stratification of species richness is incomplete without a trophic mapping of microorganism diversity, which is estimated in the trillions [42]. Nevertheless, microbiota trophic structures have been found to be functionally analogous to those of animals [49], suggesting that the trophic stratification of their species may resemble the patterns we describe.

Assuming that the observed patterns are not merely an artefact of inferring dietary preferences across regional species pools, a key question arises: why does species richness across trophic levels deviate from the pyramidal distribution across trophic levels? A pyramid of species richness would be expected based on the assumption that higher rates of biomass production at lower trophic levels support greater numbers of individuals, thereby reducing the risk of extinction and ultimately promoting higher species numbers. This idea is consistent with the ‘more individuals hypothesis’ as an explanation for species richness gradients [4,5,50]. Traditionally, the energetic structure of ecosystems has been described in terms of trophic pyramids of biomass stocks—as opposed to production rates—and often exhibits an extremely flattened pyramidal shape, with each trophic level retaining approximately 1–10% of the biomass of the level below [34,51–53]. Although biomass stocks do not perfectly capture rates of biomass production or transfer efficiency, the limited empirical evidence available suggests that energy fluxes present a similar flattened pyramidal structure across trophic levels [52,54]. Within this framework, departures from the expected pattern—such as an inverted, squared or steeply pyramidal distribution of species richness across trophic levels (figures 1 and 2)—may point to elevated diversification at higher trophic levels, beyond what would be predicted solely based on energy availability.

A non-pyramidal distribution of species richness across trophic levels presents a conceptual puzzle: how do higher-level consumers, which typically have lower population densities and hence higher extinction risks, maintain or even exceed the species richness of lower-level consumers? This pattern suggests the existence of mechanisms that either mitigate extinction risk or compensate for it through elevated speciation rates. From a biogeographical perspective, low abundances can increase population isolation, potentially promoting allopatric and parapatric speciation. If this were the dominant mechanism, we would expect higher-level consumers exhibiting smaller, more fragmented ranges than primary consumers. However, empirical evidence suggests the opposite: predators often occupy broader geographic ranges than their prey [1,55–57]. These larger ranges may, in turn, help buffer extinction risks associated with low local abundances [58,59]. Alternatively, higher trophic levels may experience greater opportunities for ecological differentiation, leading to sympatric speciation [59]. Several factors could contribute to this: the ease with which energy can be extracted from meat compared wood, which constitutes much of the primary production available for herbivores; the capacity to exploit a broader range of trophic resources (e.g. mobile versus sessile prey); the flexibility of higher-level consumers to feed across multiple trophic levels, unlike primary consumers restricted to autotrophs; and the potential for more complex interactions at higher trophic levels, where both bottom-up and top-down processes can shape niche divergence. A compelling implication of these dynamics is that higher-level consumers might experience greater species turnover across evolutionary time than herbivores—resulting from the joint effects of elevated extinction and speciation rates. Nevertheless, the wide variation in trophic configurations across taxonomic groups suggests that the diversification of predators is not governed by a universal set of processes. Instead, it is probably shaped by constraints specific to the biology, ecology and evolutionary history of each group [60]. This distinction becomes especially pertinent when comparing scavengers and detritivores with predators. While all are higher-level consumers, they differ substantially in their ecological interactions and evolutionary dynamics, particularly in their dependency on the activity or presence of other organisms to access food resources [37].

### (b) Potential drivers of uniformity in trophic structures

A separate question is why trophic structures remain remarkably uniform across regions, even though biomass pyramids become increasingly bottom-heavy in more productive ecosystems [51–53]. A plausible null expectation is that the intrinsic mechanics governing interactions among trophic levels outweigh external factors such as environmental variability or energy availability in directing the evolutionary processes that shape species’ trophic level positions [60]. Supporting this view, our results show that uniformity of trophic ratios across environments holds independently for bird and mammal communities. Moreover, the ratio of primary to higher-level consumption within the functional space of diets remains consistent across environments, even when birds and mammals are analysed separately, and despite compositional shifts in specific diet items (figure 3). Moreover, we find that trophic omnivory is consistently underrepresented across most taxa (figure 1A,B), a pattern that appears to be a general evolutionary outcome in diversification processes, as reported across diverse lineages and environments [61–64]. Such regularity in diversification patterns between trophic levels may, in part, results from the cascading effects of eco-evolutionary processes that link biodiversity across trophic levels [65–72]. Processes such as co-evolutionary dynamics, trait-mediated interactions and resource-driven diversification can propagate through food webs and shape evolutionary trajectories in interconnected ways. For example, much of plant primary production is universally allocated in wood—a resource from which it is difficult to extract energy—which may impose common constraint on the diversification of primary consumers across environments. To the extent that such processes operate consistently across regions, eco-evolutionary dynamics acting at the level of individuals could give rise to emergent regularities in trophic structure, even in the face of environmental heterogeneity.

The eco-evolutionary dynamics linking diversity across trophic levels are ultimately embedded within the broader dynamics of ecological networks. From these interactions, regional regularities in the trophic stratification of species richness may emerge. Indeed, the idea that uniformity in trophic stratification of species richness is a characteristic of local food webs has long been proposed [8,12,73,74]. Although the precise shape and universality of this pattern remain uncertain [9], the consistent positive correlation observed between predator and prey species richness at local scales suggests the existence of a general sorting mechanism that governs the distribution of species richness across trophic levels. The ecological dynamics, shaped by species interactions, influence the rates of prey and predator speciation and extinction, potentially driving food webs towards dynamic equilibria [75]. These dynamics can also shape the trophic stratification of species richness through the evolution of optimal niche breadths [37,71]. However, eco-evolutionary dynamics can also lead to alternative community states characterized by reduced biodiversity and unstable network configurations [37,71,75]. This possibility raises the intriguing prospect that uniform trophic structures may, at least in part, result from a form of selection acting at the level of complex systems—where unstable or maladaptive configurations are filtered out over time. Under this framework, general patterns may emerge not solely from deterministic processes of diversification and interaction but also from the selective retention of stable network structures [76]. Findings supporting such community-level selection dynamics include empirical evidence of changes in network properties in response to anthropogenic pressures [77], shifts in species co-occurrence patterns across regions [78] and structured responses along environmental gradients [30].

### (c) Conclusion

We show that the global species richness of terrestrial tetrapods and arthropods exhibits a non-pyramidal trophic stratification, which may ultimately hold across all terrestrial consumers. Moreover, the non-pyramidal pattern may not be limited to consumer trophic levels but could extend to the entire trophic pyramid once producers are considered. Notably, Earth’s consumers—excluding prokaryotes—account for approximately 81% of species, yet comprise only about 3% of global biomass [53,79], highlighting a striking asymmetry relative to primary producers, which dominate global biomass but account for only a small fraction of species. Additionally, the consistent ratio of species richness across trophic levels suggests that the processes shaping extinction, speciation and species sorting may be governed by universal principles. These may include system-level selection dynamics that operate alongside conventional adaptive processes centred on individual organisms. While our findings are based on a comprehensive dataset sample of over 15 000 species—including all known terrestrial birds, mammals, reptiles and amphibians—these taxa represent only a fraction of Earth’s total biodiversity. Consequently, whether globally consistent trophic structures extend across the full diversity of life remains an open question. Nevertheless, it is plausible that the eco-evolutionary forces underlying the patterns we document are not restricted to tetrapods, and may reflect broader principles of biodiversity organization. Addressing this question requires future research that explicitly tests the generality of trophic regularities across taxa and environments, and that seeks to unravel the mechanisms responsible for their emergence. Particular emphasis should be given to the roles of energy flow, interaction networks and the feedbacks between ecological and evolutionary processes. Deepening our understanding of these dynamics is crucial for informing conservation strategies that effectively address the challenges of biodiversity loss and ecosystem disruption in an era of rapid environmental change.

## 4. Methods

### (a) Global trophic structure of tetrapods and arthropods

We studied the trophic composition of the species richness of all terrestrial tetrapods and arthropods on the globe. Thus, we excluded species where aquatic (marine or freshwater) food sources involve an important proportion of their energetic input. For birds and mammals, we excluded species with marine or aquatic habits, or whose diets consisted of more than 30% fish, in order to distinguish opportunistic fish-eaters from species with predominantly aquatic diets [80–86]. For amphibians, reptiles and arthropods, we excluded species with predominantly aquatic or marine habits at any life stage [80–86], which included marine and aquatic Testudines, marine snakes, all Crocodilia, most Anura and many Caudata, and for insects all the major aquatic groups such as Ephemeroptera, Odonata, Plecoptera and Trichoptera, as well as a variety of other groups within the Coleoptera, Diptera, Hemiptera, Orthroptera, among others (for details on sources see electronic supplementary material, table S4). Thus, our analysis encompassed 5237 species of mammals, 9271 birds, 8767 reptiles, 2477 amphibians and 1 075 219 arthropods.

We classified species into three fundamental trophic levels, either as primary, higher-level or mixed-consumers, by associating numbers of species across taxa to their diet profiles as described in literature pertaining to their natural history [35]. This was done at the level of species for mammals and birds, family or lower for insects, order or lower for reptiles and amphibians and order for other arthropods. Diets and species numbers were ascribed according to descriptions in [46,84,86–114], as well as the web pages: https://www.barkbeetles.info and https://www.antwiki.org/ (for details on sources see electronic supplementary material, table S4). If more than one diet was reported for a taxon, we aimed to find the sub-taxa and number of species exhibiting each diet. Otherwise, we assumed an even distribution of each diet across species in the taxon. When predominance of species with a diet was specified (e.g. descriptions like ‘most species are’), we assumed 65–95% of species exhibited such diet (values were randomly assigned; see last paragraph of §3a for details on the implementation of this and other sources of uncertainty in the analysis), while we assumed an even distribution of the remaining species across alternative diets unless otherwise specified. If more than one diet was reported within species—rather than across species—in a taxon, we specified the percentage of the total diet comprised by each diet type following the same rules described above. Reptile and amphibian diets were classified as herbivory, carnivory and detritivory. Arthropod diets were classified as fungivory (i.e. feeding on hyphae or spores), phytophagy (i.e. feeding on any living tissue of plants, including fruits), nectivory (i.e. feeding on sap, flower nectar and pollen), predation, parasitism, ectoparasitism (i.e. feeding on blood or haemolymph), scavenging (feeding on animal carcasses), plant detritivory (feeding on dead wood or leaves) and generalist saprophagy (feeding on unspecified rotting matter). Bird and mammal diets were based on the consumption of plants, seeds, fruit, nectar, invertebrates, vertebrate endotherms, vertebrate ectotherms, fish, carrion and unknown vertebrates [80–86].

We used diet information to calculate the percentage of primary and higher-level consumption of species across taxa. Phytophagy and nectivory were classified as primary-level consumption and predation, parasitism and ectoparasitism as higher-level consumption. We classified fungivory as higher-level consumption because fungi are themselves primary consumers. Plant detritivory was classified as 100% to 50% primary-level consumption as it is unknown how much of this diet derives energy from the plant biomass or from fungi consuming the plant biomass. General detritivory and saprophagy was classified as 100% to 0% primary-level consumption, as it is unknown how much of this diet derives energy from algal, bacterial, plant, fungal or animal matter. We classified scavenging as higher-level consumption as whether an animal’s prey is dead or alive bears no importance to its trophic status as a consumer [34]. We considered kleptoparasitizing, dung eating, and honeydew consumption as higher-level consumption (i.e. within scavenging) as these feeding strategies rely on products of animal consumers [115].

For taxa with metamorphosis (e.g. holometabolous insects), we conducted a differential analysis of diet composition between developmental stages and averaged them. In many insect taxa the bulk of energetic consumption and biomass accumulation occurs in the larval stage, while adults exhibit an exclusive nectivorous diet providing the minimum necessary nourishment [116]. Thus, for insect taxa with exclusively nectivorous adults, we limited adult diet weight from 10% to 50% when averaging with the larva diet (values were randomly assigned; see next paragraph for details on this and other sources of uncertainty).

We classified species according to their trophic level either as primary consumers (i.e. more than 2/3 of diet consists of primary consumption), higher-level consumers (i.e. more than 2/3 of diet consists of higher-level consumption) and mixed consumers (i.e. between 1/3 and 2/3 of diet consists of primary or higher-level consumption). We addressed uncertainty regarding the proportion of predominant diets across and within taxa, the proportion of primary consumption involved in general saprophagy and detritivory, and the energetic weight that adults represent in insect taxa with metamorphosis by randomizing these values in 1000 trophic pyramids.

### (b) Trophic structures of tetrapods across the globe

We studied the trophic composition of terrestrial tetrapod communities (i.e. birds, mammals, reptiles and amphibians) around the world by combining data on species’ trophic level—derived from the analysis described in the previous section—with data on species’ native ranges derived from the IUCN Global Assessment [117,118]. This included trophic level data for 3694 mammal, 6270 bird, 5418 reptile and 752 amphibian species across a global grid of 1 degree pixels. We build on a framework that identifies six distinct community trophic structures analogous to trophic biomes (i.e. depauperate, boreal, temperate, semi-arid, seasonal tropical and humid tropical), which are defined by varying proportions of species in different guilds across regions with different levels of ecosystem productivity [30–33] (electronic supplementary material, figure S1). We compare the trophic stratification of species richness across these six community trophic structures based on the proportion of species in different trophic levels, rather than the proportion in different guilds as originally defined. For each pixel, we calculated the proportion of primary, mixed and higher trophic-level species in the community. We then used a discriminant analysis to assess the degree of differentiation in the trophic composition between the six guild configuration patterns, where the proportions (arcsine(√*x*) transformed) of primary, mixed and higher-level consumer species in each pixel (*n* = 14 496) served as response variables (electronic supplementary material, table S3). This analysis was replicated for bird and mammal communities independently, but not for reptiles and amphibians, as these are almost entirely composed of higher-level consumers, leaving little room for variation across the six trophic structures.

Finally, we took advantage of detailed diet data for mammals and birds to assess the contribution of different diet elements to the aggregated diet of the community in each pixel, and compare them across the six trophic structures. Thus, for the bird and mammal community in each pixel, we added the percentages of different elements (i.e. plants, seeds, fruit, nectar, invertebrates, vertebrate endotherms, vertebrate ectotherms, fish, carrion and unknown vertebrates) constituting the diet of each species and then calculated the ratio of primary (i.e. plant-based) to higher-level consumption (i.e. animal-based) in the aggregated diet of the community. We used an analysis of variance (ANOVA) to compare the percentage of primary consumption (as opposed to higher-level consumption) between pixels of each of the six trophic structures, and used the *R*^2^ to assess their explanatory power. This analysis was replicated for bird and mammal communities independently.

Analyses were carried out in *R* [119]. We used packages *dplyr* for data management [120], *ggplot2* [121], *ggpubr* [122] and *lemon* [123], for figure design. The data and code that support the findings of this study are openly available at https://doi.org/10.6084/m9.figshare.27104014.v1 [124].

## Data Availability

The data and code that support the findings of this study are openly available at [124]. Supplementary material is available online [125].
